# Prospective study of clinical and laboratorial hypocalcemia after thyroid surgery

**DOI:** 10.1590/S1808-86942010000100012

**Published:** 2015-10-17

**Authors:** Rogério Aparecido Dedivitis, Elio Gilberto Pfuetzenreiter, Carlos Eduardo Molinari Nardi, Emmanuel Casotti Duque de Barbara

**Affiliations:** 1 Professor at Lusíada Foundation UNILUS, MD; 2 MD, Head and Neck Surgeon, Ana Costa Hospital, Santos; 3 MD, Resident of General Surgery, Ana Costa Hospital, Santos; 4 MD, Former intern at the Ana Costa Hospital, Santos; 5 Graduate Program on Health Sciences, Heliópolis Hospital HOSPHEL, São Paulo; Service of Head and Neck Surgery, Ana Costa Hospital, Santos; Program of Head and Neck Surgery, Lusíada Foundation UNILUS; Service of Head and Neck Surgery, Irmandade da Santa Casa da Misericórdia de Santos

**Keywords:** postoperative complications, thyroid diseases, parathyroid glands, hypocalcemia, thyroidectomy

## Abstract

Hypocalcemia can be detected clinically and through lab tests after thyroidectomy.

**Aim:**

To analyze the incidence and risk factors of clinical and laboratorial hypocalcemia after thyroid surgery.

**Methods:**

Prospective study of 91 patients undergoing thyroidectomy. Demographics, intraoperative, and pathological aspects were correlated to our hypocalcemia findings.

**Results:**

Age higher than 50 (p = 0.022) and complete thyroidectomy (p < 0.001) were considered risk factors for hypoparathyroidism. Complete thyroidectomy was considered a risk factor for the 48-hour laboratorial hypoparathyroidism (p = 0.004). There was no risk factor associated with the one-month laboratorial hypoparathyroidism. There was significance between the 48-hour and the one-month laboratorial hypoparathyroidism.

**Conclusions:**

Thyroidectomy extension is a risk factor for both the clinical and laboratorial hypoparathyroidism, whereas age is a risk factor for clinical hypoparathyroidism. The detection of 48-hour laboratorial hypoparathyroidism is a predisposing factor for the one-month laboratorial hypoparathyroidism. However, most of the cases were temporary.

## INTRODUCTION

Post-thyroidectomy hypocalcemia is a serious early complication. Patients must therefore be carefully observed in the postoperative period and have their lab workup done, especially those categorized as high risk patients^1^. Estimates indicate that transient hyperparathyroidism prevalence rates range between 6.9% and 46% and permanent hyperparathyroidism rates vary from 0.4% to 33%^2^, ^3^, ^4^, ^5^.

Hypocalcemia may occur secondarily to surgical trauma, devascularization, unintentional removal of parathyroid glands, reoperation, and total thyroidectomy^6^. Even after meticulously performed procedures, some temporary parathyroid dysfunction may occur^7^. Surgery extension has been seen as a risk factor, as in total thyroidectomy there is potential blood supply involvement resulting from bilateral surgical manipulation. Lobectomy patients are at extremely low risk of postoperative hypocalcemia, as parathyroid glands usually remain functional at the non-operated side^1^.

Some situations are considered to be risk factors for the onset of transient and permanent hyperparathyroidism, such as Graves' disease, recurring goiter, and thyroid carcinoma. However, other factors are related to the chosen surgical procedure and its impact on devascularization or accidental removal of the parathyroid glands. The recommended surgical strategy is meticulous dissection and preservation of the parathyroid glands and their blood supply^5^. While the removal of one gland is not associated with postoperative hypocalcemia, the same cannot be said when two or more glands are removed^4^. The best way to avoid accidental excision is properly identifying the parathyroid glands. Risk of complication is higher when fewer than three glands are identified during surgery^4,^^8,^^9^.

This paper aims to prospectively study the possible risk factors for the onset of post-thyroidectomy hypocalcemia.

## MATERIALS AND METHOD

Our study enrolled 91 patients who had their thyroid glands surgically treated from July to December of 2007. This is an observation study with prospectively collected data. All patients were tested preoperatively, 48 hours into postoperative care and 30 days after surgery for serum ionic calcium by the same laboratory. None of the patients had undergone thyroid surgery or radiotherapy before, and none of them was lost during follow-up. All patients had preoperative calcium levels within normal ranges, and none were undergoing calcium or vitamin D replacement. This study was approved by the Ethics Committee at our institution. The study aimed at correlating the demographic, intraoperative, and pathologic risk factors mentioned above to early (48 hours) and late (one month) hypocalcemia.

The following parameters were found:

- demographic factors: gender; age; clinical diagnosis (uninodular goiter, multinodular goiter, or thyroid cancer);
- technical surgical aspects: surgery type (partial thyroidectomy - lobectomy with isthmectomy, total thyroidectomy and total thyroidectomy with neck clearance); and number of parathyroid glands reinserted into the sternocleidomastoid muscle after unintentional excision;
- pathology aspects: volume of the thyroid lobe involved by lesion or the dominant one; volume of contralateral lobe; gland weight (in grams); pathology test results (goiter, adenoma, or thyroid cancer); and thyroiditis (according to pathology parameters). The data sets were analyzed in accordance with the incidence of clinical and laboratory hypoparathyroidism (at 48 hours and one month into postoperative care) (Table 1).

**Table 1:** Patient distribution according to demographic variables.

Variable	Categories / Measurements	Freq (%) / Measurements
Gender	Male	7 (7,7)

	Female	84 (92,3)
Age (years)	N	91
	Minimum-maximum	18–82
	Median	50
	Mean	49,3
	Standard deviation	14,0
Clinical Diagnosis	NTUG	39 (42,9)
	NTMG	40 (44,0)
	Cancer	12 (13,2)
Procedure	Partial	45 (49,4)
	TT	44 (48,4)
	TT + NC	2 (2,2)
Weight	N	91
	Minimum-maximum	6–320
	Median	25
	Mean	36,6
	Standard deviation	40,3
Pathology tests	Goiter	51 (56,0)
	Adenoma	19 (20,9)
	Cancer	21 (23,1)
Thyroiditis	No	46 (50,6)
	Yes	45 (49,4)
Clinical HPT	No	80 (87,9)
	Yes	11 (12,1)
Laboratorial HPT at 48h	No	69 (75,8)
	Yes	22 (24,2)
Laboratorial HPT at 1 month	No	85 (93,4)
	Yes	6 (6,6)

TT = total thyroidectomy; NC = neck clearance; HPT = hypoparathyroidism

The chi-square test was used to verify the association between categorical variables in contingency Tables. Fisher's exact test was applied in 2x2 Tables in which at least one expected frequency is lower than 5. The association between numeric variables in relation to the occurrence of hypoparathyroidism at one month and 48 hours was verified by McNemar's test. A significance level of 5% was adopted or all statistical tests.

## RESULTS

The following were the risk factors considered for the onset of clinical hypoparathyroidism (Table 2): age above 50 years (p = 0.022) and non-partial surgery (total thyroidectomy and total thyroidectomy with neck clearance) - p < 0.001. Non-partial surgery (p=0.004) was identified as a risk factor for the onset of laboratorial hypoparathyroidism detected through calcium serum levels 48 hours into postoperative care (Table 3). No risk factors were found for the onset of laboratorial hypoparathyroidism one month into follow-up (Table 4). Only one patient had parathyroid insertion secondary to accidental gland excision confirmed through frozen sample testing on the bed of the sternocleidomastoid muscle.

**Table 2:** Patient distribution according to clinical hypoparathyroidism.

Variable	Categories	Clinical HPT		p-value
		NO N(%)	YES N(%)	
Gender	Male	7 (100,0)	0 (0,0)	0,592
	Female	73 (86,9)	11 (13,1)	
Age range (years)	< 50	44 (95,6)	2 (4,4)	0,022*
	> 50	36 (80,0)	9 (20,0)	
Clinical diagnosis	NTUG	38 (97,4)	1 (2,6)	N/A
	NTMG	32 (80,0)	8 (20,0)	
	Cancer	10 (83,3)	2 (16,7)	
Diagnosed with Multinodular Goiter	Non-NTMG	48 (94,1)	3 (5,9)	0,054
	NTMG	32 (80,0)	8 (20,0)	
Procedure	Partial	45 (100,0)	0 (0,0)	N/A
	TT	33 (75,0)	11 (20,0)	
	TT + NC	2 (100,0)	0 (0,0)	
Partial procedure	Partial	45 (100,0)	0 (0,0)	< 0,001*
	Non-partial	35 (76,1)	11 (23,9)	
Weight	< 25	43 (89,6)	5 (10,4)	0,605*
	> 25	37 (86,0)	6 (14,0)	
Pathology tests	Goiter	44 (86,3)	7 (13,7)	N/A
	Adenoma	18 (94,7)	1 (5,3)	
	Cancer	18 (85,7)	3 (14,3)	
Thyroiditis	No	42 (91,3)	4 (8,7)	0,316
	Yes	38 (84,4)	7 (15,6)	

p-value obtained through Fisher's exact test; * p-value obtained through chi-square test; N/A = not applicable; NTUG = nontoxic uninodular goiter; NTMG = nontoxic multinodular goiter; TT = total thyroidectomy; NC = neck clearance.

**Table 3:** Patient distribution according to laboratorial hypoparathyroidism at 48 hours.

Variable	Categories	Laboratorial HPT at 48h		p-value
		NO N(%)	YES N(%)	
Gender	Male	7 (100,0)	0 (0,0)	0,189
	Female	62 (73,8)	22 (26,2)	
Age range (years)	< 50	38 (82,6)	8 (17,4)	0,126*
	> 50	31 (68,9)	14 (31,1)	
Clinical diagnosis	NTUG	33 (84,6)	6 (15,4)	N/A
	NTMG	27 (67,5)	13 (32,5)	
	Cancer	9 (75,0)	3 (25,0)	
Diagnosed with NTMG	Non-NTMG	42 (82,4)	9 (17,6)	0,100
	NTMG	27 (67,5)	13 (32,5)	
Procedure	Partial	40 (88,9)	5 (11,1)	N/A
	TT	27 (61,4)	17 (38,6)	
	TT+NC	2 (100,0)	0 (0,0)	
Partial Procedure	Partial	40 (88,9)	5 (11,1)	0,004*
	Non-partial	29 (63,0)	17 (37,0)	
Weight	< 25	38 (79,2)	10 (20,8)	0,431*
	> 25	31 (72,1)	12 (27,9)	
Pathology tests	Goiter	37 (72,6)	14 (27,4)	N/A
	Adenoma	16 (84,2)	(15,8)	
	Cancer	16 (76,2)	5 (23,8)	
Thyroiditis	No	38 (82,6)	8 (17,4)	0,126
	Yes	31 (68,9)	14 (31,1)	

p-value obtained through Fisher's exact test; * p-value obtained through chi-square test; N/A = not applicable; NTUG = nontoxic uninodular goiter; NTMG = nontoxic multinodular goiter; TT = total thyroidectomy; NC = neck clearance.

**Table 4:** Distribution according to laboratorial hypoparathyroidism at one month.

Variable	Categories	Laboratorial HPT at 1 month		p-value
		NO N(%)	YES N(%)	
Gender	Male	7 (100,0)	0 (0,0)	> 0,999
	Female	78 (92,9)	6 (7,1)	
Age range (years)	< 50	42 (91,3)	4 (8,7)	0,677
	> 50	43 (95,6)	2 (4,4)	
Clinical diagnosis	NTUG	38 (97,4)	1 (2,6)	N/A
	NTMG	36 (90,0)	4 (10,0)	
	Cancer	11 (91,7)	1 (8,3)	
Diagnosed with NTMG	Non-NTMG	49 (96,1) 3	2 (3,9)	0,399
	NTMG	6 (90,0)	4 (10,0)	
Procedure	Partial	44 (97,8)	1 (2,2)	N/A
	TT	39 (88,6)	5 (11,4)	
	TT+NC	2 (100,0)	0 (0,0)	
Partial Procedure	Partial	44 (97,8)	1 (2,2)	0,203
	Non-partial	41 (89,1)	5 (10,9)	
Weight	< 25	45 (93,8)	3 (6,2)	> 0,999
	> 25	40 (93,0)	3 (7,0)	
Pathology tests	Goiter	48 (94,1)	3 (5,9)	N/A
	Adenoma	17 (89,5)	2 (10,5)	
	Cancer	20 (95,2)	1 (4,8)	
Thyroiditis	No	43 (93,5)	3 (6,5)	> 0,999
	Yes	42 (93,3)	3 (6,7)	

p-value obtained through Fisher's exact test; N/A = not applicable; NTUG = nontoxic uninodular goiter; NTMG = nontoxic multinodular goiter; TT = total thyroidectomy; NC = neck clearance.

Table 5 shows statistical significance between laboratorial hypoparathyroidism at 48 hours and one month into follow-up; 16 patients had hypoparathyroidism at 48 hours and ten ceased to have it at one month into follow-up.

**Table 5:** Distribution according to laboratorial hypoparathyroidism at 48 hours and one month.

Variable	Categories	Laboratorial HPT at 1 month		p-value
		NO N(%)	YES N(%)	
Laboratorial HPT at 48	No	69 (100,0)	0 (0,0)	< 0,001
	Yes	16 (72,7)	6 (27,3)	

p-value obtained from McNemar's test

## DISCUSSION

Transient hypocalcemia is commonly seen after thyroid surgery, as biochemical disorders are described in more than 83% of the cases^6,^^7,^^10,^^11^; clinical hypocalcemia is however less frequently observed.

Thyroid surgery extension was reported as an important factor in the prevalence rates of transient and permanent hypoparathyroidism when more and less extensive procedures were compared^10^; in multivariate analysis this was found to be the most important isolated factor^5^. Some authors have found that patients undergoing unilateral surgery do not present signs of clinical or laboratorial hypocalcemia^1^,^6^. On the other hand, for patients submitted to total thyroidectomy there is a potential risk of vascular involvement of the four parathyroid glands due to bilateral manipulation^1^. In our study, the performance of a non-partial thyroidectomy - that is, total thyroidectomy or total thyroidectomy with neck clearance - was a risk factor for the onset of both clinical and laboratorial hypoparathyroidism. Only two patients required total thyroidectomy with neck clearance (Table 1) and neither of these patients presented clinical or laboratorial hypoparathyroidism. Therefore, in our series neck clearance cannot be considered as a risk factor for the analyzed outcomes.

In a consecutive series of 200 patients submitted to thyroidectomy for nontoxic multinodular goiter, the risk of postoperative hypoparathyroidism was 25 times greater for patients above the age of 50, 28 times greater for preoperative levels of 25-vitamin D lower than 15 ng/mL and 71 times greater for patients submitted to total thyroidectomy^12^.

Many authors (us included) prefer to ligate the inferior thyroid artery peripherally, i.e., close to the thyroid capsule^13^, ^14^, ^15^. Such procedure, instead of ligating the ITA close to the branch origin, would better preserve the integrity of the parathyroid branches. Although a randomized trial with 100 patients showed no significant differences in postoperative hypoparathyroidism between central and peripheral ligation^16^, a vast series analyzed through multivariate analysis showed that bilateral central ligation is an independent factor for hypoparathyroidism, mainly for permanent hypoparathyroidism^5^. As a rule, we perform peripheral ligations. This occurred in all cases in our series, thus rendering impossible a statistical comparison against central ligation.

It is recommended that parathyroid glands are spared based on careful dissection of the gland's blood pedicle. Permanent hypoparathyroidism occurs exclusively when fewer than three parathyroid glands are identified during surgery^8,^^9,^^11^. Other authors consider that the identification and sparing of at least two glands may result in higher rates of permanent hypoparathyroidism^5^. This parameter was not assessed in our study, as in cases of partial thyroidectomy in which only one side is explored the number of identified glands is naturally lower, as the contralateral side has not been dissected and significant biases would be introduced as a result of such measure.

Considering unintentional lesions and avulsions, parathyroid tissue is often found in excised thyroid specimens even when exploratory dissection to search for parathyroid glands is performed by experienced surgeons^17^.

Various methods have been proposed to identify parathyroid glands in the site of surgery. Esselstyn, in more than 200 cases of neck exploration, removed about a third of the gland after locating it in the contralateral side on the vascular pedicle and sent them for pathology testing. Macroscopic surgical confirmation of the parathyroid gland was performed through observation, in the first three seconds, of a red dots appearing in the sectioned gland surface (parathyroid blush)^18^. Silverberg studied 19 cases of hyperparathyroidism through the imprint technique on a glass slide using methylene blue staining, achieving 100% accuracy in the 96 studied specimens to differentiate thyroid, parathyroid, and lymph node tissue. The advantage elicited by the author is the short time - 45 seconds to one minute - required to perform the diagnosis^19^. Romão, in an experimental trial, used intravenous methylene blue during surgery in ten dogs and intrarterial in eight dogs. The intrarterial methylene blue injections failed to show the location of the glands; stain leakage and bleeding were additionally observed. Glands were more easily identified in the group of intravenous injections^20^. Kuhel and Carew (1999) conducted a prospective study in 14 thyroidectomy procedures and used incisional biopsy - 34 in 36 possible glands were confirmed. In 17 (50%) cases, there was bleeding as expected from the remainder of the gland at the site where the biopsy was performed. All others were deemed as devascularized, whether or not their color was altered, and were reimplanted in the sternocleidomastoid muscle. The authors attributed the lack of bleeding during incisional biopsy as a criterion for devascularization, as it is more reliable than color alteration^21^. Perrier et al. carried out a study using fine needle aspiration during surgery to biochemically quantiy the presence of parathyroid hormone in 65 subjects; all aspiration findings from tissues such as the thyroid gland, fat tissue, lymph node, and thymus presented ten times less parathyroid hormone when compared to the values found in the parathyroid gland, with high sensitivity and specificity^22^. Sofola et al. studied four rabbits using orthogonal polarized spectral imaging. The device worked as an 'intravital' microscope to find the parathyroid glands. The image obtained from tissue microvasculature architecture made it possible to characterize each tissue individually, differentiating fat tissue from thyroid, and parathyroid glands^23^. Shidham et al. (2002) prospectively analyzed the use of cytology tests through scraping of 177 fresh specimens obtained during parathyroidectomy and concluded that the concurrent use of this method with frozen biopsy is the best means for gland identification^24^. Pedersonet al. used a portable gamma camera in 13 patients undergoing neck central compartment surgery to identify and preserve parathyroid glands, using sestamibi as the radio-tracing agent. In six cases the radioisotope was found in the surgical specimen, allowing for prompt reimplantation^25^. Yao et al. performed a double-blind retrospective study in which five pathologists re-examined 50 biopsy and TIP (touch imprint preparation) products of supposedly parathyroid tissue obtained during thyroidectomy. Diagnosis was unanimous in 33 (66%) cases. The authors concluded that the technical problems commonly seen on TIP were associated to difficulties in interpretation and technical preparation^26^. Rohaizak et al. conducted a prospective cytology intraoperative study through tissue scraping and the technique identified 25 out of 29 glands, with 88.2% accuracy and 100% specificity^27^. Guimarães prospectively studied 125 patients through contact endoscopy; 331 glands were found by surgeons and 282 by endoscopy. Nine (7.2%) PT were found among surgical specimens. The Kappa correlation index comparing the findings made by surgeons and contact endoscopy was of 0.534. The longer it took for surgery to be completed (p=0.03) and the time to locate and identify the gland (p=0.00), the smaller was the observed agreement. As the use of endoscopy entered its second year, greater agreement was found (p=0.02). All 17 autotransplanted glands were identified through contact endoscopy and confirmed through frozen biopsy^28^. Grubb-set al. used a gamma probe to identify parathyroid glands tagged with sestamibi during neck central compartment surgery in 54 patients; the method was useful in 20 cases, nine of which in vivo and 11 in surgical specimens in which the gland had been accidentally removed. This method was particularly recommended in cases of reoperation^29^.

Parathyroid removal was seen in 17.7% of the patients submitted to thyroidectomy; in 86% of the cases the removal was unintentional. In this case or when gland vasculature is compromised, autoimplantation is strongly recommended instead of in situ preservation, mainly when two or more parathyroid glands are involved^7^. When there is doubt as to the nature of the tissue, frozen sections can be analyzed for confirmation. This technique consists of slicing gland tissue in 1mm fragments (usually resulting in 10 to 20 specimens) and inserting them in individual pouches within the ipsilateral sternocleidomastoid muscle, followed by suturing with non-absorbing thread on the muscle after autoimplantation^30^.

The experience of the surgical team was shown to be a risk factor in a multicenter study: the greater the volume of surgery the lower was the rate of hypoparathyroidism^31^.

An interesting finding in our series was the statistical significance encountered between laboratorial hypoparathyroidism detection within 48 hours and one month into follow-up. This finding suggests the need for oral calcium replacement when complications are found early on. On the other hand, when tests are normal after 48 hours, none of the patients evolved to hypoparathyroidism at a later time.

## CONCLUSION

The extension of the thyroid resection procedure is a risk factor for the onset of clinical and laboratorial hypoparathyroidism, while age above 50 years is a risk factor for clinical hypoparathyroidism. Laboratorial hypoparathyroidism detected at 48 hours into follow-up is a predisposing factor for laboratorial hypoparathyroidism one month into postoperative care. On the other hand, 10 of the 16 patients with hypoparathyroidism at 48 hours had normal serum ionic calcium levels one month into postoperative care, characterizing the transient nature of this disease in most of the cases.
